# Supplementary material for: Plant-Assisted Synthesis of Ag-Based Nanoparticles on Cotton: Antimicrobial and Cytotoxicity Studies
Source: Molecules. 2024 Mar 23;29(7):1447. doi: 10.3390/molecules29071447 (PMC11013149; doi:10.3390/molecules29071447)
Supplement: Supplementary file 1 [file molecules-29-01447-s001.zip › molecules-2888873-supplementary.pdf]

Table S1 Color coordinates of impregnated fabrics*

Sample	Color coordinates							
	L^*	a^*	b^*	C^*	h	X	Y	Z
CO-CA-Ag-PSC	47.34	16.32	21.35	26.87	52.62	18.37	16.28	9.10
CO-CA-Ag-JR	35.22	15.19	17.91	23.48	49.70	9.97	8.61	4.68
CO-CA-Ag-HL	73.20	10.05	9.42	13.78	43.14	46.57	45.46	40.36
CO-CA-Ag-SN	45.22	13.63	11.19	17.64	39.38	16.21	14.70	11.27

*Color coordinates are determined by Datacolor SF300 spectrophotometer under illuminant D_{65} using the 10° standard observer, where *L** stands for lightness, *a** red/green value and *b** yellow/blue value.
